# Development and application of a high-sensitivity immunochromatographic test strip for detecting pseudorabies virus

**DOI:** 10.3389/fmicb.2024.1399123

**Published:** 2024-05-03

**Authors:** Jiajia Yin, Huimin Liu, Yumei Chen, Jingming Zhou, Yankai Liu, Zhenglun Liang, Xifang Zhu, Hongliang Liu, Peiyang Ding, Enping Liu, Ying Zhang, Sixuan Wu, Aiping Wang

**Affiliations:** ^1^Longhu Laboratory, Zhengzhou, China; ^2^School of Life Sciences, Zhengzhou University, Zhengzhou, China; ^3^College of Basic Science, Zhengzhou University of Technology, Zhengzhou, Henan, China; ^4^Henan Provincial Key Laboratory of Immunobiology, Zhengzhou, China

**Keywords:** pseudorabies virus, monoclonal antibody, gold nanoparticles, immunochromatographic strip, rapid antigen testing

## Abstract

**Introduction:**

Pseudorabies (PR) is a multi-animal comorbid disease caused by pseudorabies virus (PRV), which are naturally found in pigs. At the end of 2011, the emergence of PRV variant strains in many provinces in China had caused huge economic losses to pig farms. Rapid detection diagnosis of pigs infected with the PRV variant helps prevent outbreaks of PR. The immunochromatography test strip with colloidal gold nanoparticles is often used in clinical testing due to its low cost and high throughput.

**Methods:**

This study was designed to produce monoclonal antibodies targeting PRV through immunization of mice using the eukaryotic system to express the gE glycoprotein. Subsequently, paired monoclonal antibodies were screened based on their sensitivity and specificity for use in the preparation of test strips.

**Results and discussion:**

The strip prepared in this study was highly specific, only PRV was detected, and there was no cross-reactivity with glycoprotein gB, glycoprotein gC, glycoprotein gD, and glycoprotein gE of herpes simplex virus and varicellazoster virus, porcine epidemic diarrhea virus, Senecavirus A, classical swine fever virus, porcine reproductive and respiratory syndrome virus, and porcine parvovirus. Moreover, it demonstrated high sensitivity with a detection limit of 1.336 × 10^3^ copies/μL (the number of viral genome copies per microliter); the coincidence rate with the RT-PCR detection method was 96.4%. The strip developed by our laboratory provides an effective method for monitoring PRV infection and controlling of PR vaccine quality.

## Introduction

Pseudorabies (PR), also known as Aujeszky disease (AD), is a highly contagious viral disease caused by the pseudorabies virus (PRV), which can infect a variety of animals (Wong et al., 2019; Yu et al., 2021). PRV was first found in cats in China in 1957, and it spread to many regions in China in the 1980s, infecting a variety of animals. Pigs are natural hosts for PRV and can carry the virus latently (Yu et al., 2014). China, known as the largest producer and consumer of pork, has encountered two instances of pseudorabies outbreaks. The first outbreak occurred in the 1990s and then again in 2011 (Dong et al., 2018). Experiments such as virus isolation proved that the root cause of the 2011 outbreak was the mutation of the strain. The PRV variant strains when compared to classical strains exhibited a higher level of virulence in pigs. While the traditional Bartha-K61 vaccine strain can provide comprehensive protection against the classical strains, it proves inadequate in defending against variant strains (Meng et al., 2016; Cheng et al., 2020; Tan et al., 2021). PRV underwent mutation and regained its virulence, leading to a widespread outbreak in China. Henan, a major province for pig farming, has been particularly affected by this epidemic. The recirculation of pseudorabies wild-type poison in Chinese pig farms has posed great challenges to both the pig industry and efforts toward preventing and controlling epidemic diseases (Tan et al., 2021).

Identification, rapid eradication, and vaccination of infected pigs are crucial measures implemented currently for controlling pseudorabies in the porcine population. Currently, the predominant clinical approaches for PRV diagnosis primarily involve serological detection and molecular biology techniques. Molecular biology detection methods encompass fluorescence quantitative PCR and reverse transcription PCR (RT-PCR) (Cheng et al., 2021). However, the use of PCR for diagnosing PRV infection in pigs is limited due to its requirement for specialized laboratory personnel (Porte et al., 2020). Serological detection methods for PRV mainly include microneutralization assay (MN), indirect enzyme-linked immunosorbent assay (i-ELISA), competitive ELISA (c-ELISA), and indirect immunofluorescence assay (IFA). Among these methods, blocking ELISA is commonly employed due to its high specificity and sensitivity, but is time-consuming (Maan et al., 2012).

Rapid antigen (RAD) testing plays a crucial role in the early diagnosis and containment of diseases, offering potential as an important diagnostic tool for PRV, particularly when molecular methods of detection are limited (Mak et al., 2020; Scohy et al., 2020). Additionally, RAD can also be utilized to monitor whether there are residues of gE antigens in vaccines. In recent years, there has been an increase in the severity of co-infections between PR and other swine diseases, and there is an urgent need for rapid and accurate detection of PR antigens. The immunochromatography test strip rapid technology is an immunological rapid detection method developed based on the mAb technology, which has become a primary approach for rapid detection. The antigen test strip commonly adopts the “double antibody sandwich” detection mode to enable the identification of microbial antigens such as viruses, bacteria, and parasites, along with non-microbial antigens including self-antigens and tumor antigens (Wang et al., 2015). Therefore, this study aims to develop immunochromatographic strips for PR screening.

At present, a total of 11 glycoproteins have been identified in porcine pseudorabies virus: gB (UL27), gC (UL44), gD (US6), gE (US8), gG (US4), gH (UL22), gI (US7), gK (UL53), gL (UL1), gM (UL10), and gN (UL49.5). The gE protein encoded by US8 plays a crucial role in virulence, and its absence reduces the virus's infectivity to the host (Tirabassi and Enquist, 1999). Therefore, gE-deleted strains are commonly used in vaccine research. Additionally, gE is frequently used as a marker target in detection methods for differentiating between vaccination and wild-type infection (Wang et al., 2018). The advantage of using the gE protein as a marker to distinguish between antibodies resulting from wild-type virus infection and those induced by vaccination lies in its ubiquitous expression across all wild-type virus strains (van Oirschot et al., 1990; Tirabassi et al., 1997), thereby enabling the establishment of PRV antigen detection methods based on this characteristic.

In this study, mice were immunized with the gE protein used as an immunogen to generate monoclonal antibodies (mAbs) against PRV. Subsequently, a gold immunochromatographic strip specific for PRV was developed using two gE-specific mAbs, with the PRV gE protein having a detection limit of 31.25 ng/mL. The immunoassay strip described in this article enables rapid detection of PRV gE protein, making it suitable for early infection diagnosis of wild-type PRV strains and quality monitoring of PRV vaccines.

## Materials and methods

### Ethics and biosafety statements

The experimental research protocol for monoclonal antibody production in mice was approved by the Key Laboratory of Animal Immunology, Henan Academy of Agricultural Sciences, China, in line with its policies and procedures (LLSC100166).

### Reagent, strains, and cells

DH5α competent cells were purchased from Biomedical Technology (Beijing, China). FreeStyle™ 293-F cells were sourced from Sino Biological (Beijing, China). SP2/0 cells were provided by the Key Laboratory of Animal Immunology of Henan Academy of Agricultural Sciences (Zhengzhou, China). 293-F cells were cultured using SMM 293-TII serum-free medium (Sino Biological, China). SP2/0 cells were maintained with RPMI 1640 medium (Solarbio, China), supplemented with 10% (v/v) fetal bovine serum (TransGen, Beijing). Aminophenylboronate A6XL was purchased from Astrea Bioseparations Co., Ltd. (Cambridge, UK). Superdex200 10/300 GL was purchased from Cytiva (Montana, USA). The virus strain HeNL/2017 (GenBank no. MT775883) recombinant PRV-GFP strain was kindly provided by researcher Wang Hanzhong from Wuhan Institute of Virology, Chinese Academy of Sciences. The virus strain HeNL/2017 was propagated with DMEM supplemented with 2% (v/v) fetal bovine serum. Porcine epidemic diarrhea virus (PEDV), Senecavirus A (SVA), classical swine fever virus (CSFV), porcine reproductive and respiratory syndrome virus (PRRSV), and porcine parvovirus (PPV) were preserved and propagated by this experiment.

### Expression and purification of PRV gE

The genomic sequence of HeNLH/2017 (GenBank:QQL12221.1) was used as a sequence template to construct the gE protein, which was subsequently optimized based on a codon usage bias of mammalian expression systems and synthesized by General Biotech Co., Ltd. (Anhui, China). Following the In-Fusion^®^HD Cloning Kit manual for inserting gE into the pCAGGS plasmid (HonorGene, China), the recombinant plasmid was transfected into 293F cells mediated by polyethylenimine linear (PEI) MW40000 (Yisheng, China) with a cell density of 2~3 × 10^6^ cells/mL. After 72 h of transfection, the supernatant was harvested by centrifugation at 12,000 g × 10 min at 4°C and then identified by Western blot.

The supernatant was filtered through a 0.22-μm Stericup filter and purified by aminophenylboronate A6XL affinity chromatography. Subsequently, the eluted protein solution was dialyzed against phosphate buffer (PBS, pH 7.4). The dialyzed protein solution was concentrated by using an ultrafiltration tube (MilliporeSigma, USA) with a retention capacity of 30 K and loaded onto the Superdex200 gel filtration chromatography column to separate the recombinant protein and identified by SDS-PAGE and Western blot. The concentration of the recombinant gE protein was determined using a NanoDrop2000 spectrophotometer (Thermo Fisher Scientific, USA).

### Preparation of monoclonal antibodies against PRV gE

A standard protocol was followed to generate mAbs against PRV gE. Briefly, 6-week-old female Balb/c mice (*n* = 2) were immunized with PRV gE protein at a dose of 10 μg per mouse in Freund's adjuvant medium. Blood samples were collected 14 days after the third immunization. The serum titer level was determined through indirect ELISA with the recombinant gE protein as a coated antigen. Mice with high serum titers were selected for the preparation of hybridoma cells. Positive hybridoma cell lines were screened, and the supernatant of positive hybridoma cell lines was identified by ELISA. Positive hybridoma cell lines were diluted through limiting dilution to obtain a monoclonal cell line that stably produced antibodies. IFA was used to further verify the reactivity of the mAbs against the gE protein. Ascitic fluids from positive hybridomas for colloidal gold labeling were produced in mice.

### Immunofluorescence assay

The HEK293T cells were cultured in DMEM supplemented with 10% FBS and seeded into 48-well cell culture plates at a density of 2 × 10^4^ cells/well. They were then incubated overnight at 37°C with 5% CO_2_. Once the cells reached a confluency of 70–80%, the recombinant plasmid pCAGGS-gE was transfected into the cells using the jetPRIME^®^ Versatile DNA/siRNA transfection reagent (Polyplus, France) and cultured at 37°C for 48 h. After culturing, the plates were fixed with methanol (precooled to −20°C) for 20 min at room temperature (RT). Next, the plates were washed with PBST and incubated with skim milk (5%) at 37°C for 1 h. Then, anti-PRV gE was used as a primary antibody for 30 min, while DMEM served as a negative control. Subsequently, the plates were washed three times with PBST and incubated with anti-mouse IgG H&L (FITC) (Solarbio, Beijing, China) for half an hour at 37°C. Cells were simultaneously stained using DAPI (4′,6-diamidino-2-phenylindole) from Solarbio. After the final washing, fluorescence signals were visualized using fluorescence microscopy from ZEISS in Jena, Germany.

### Preparation of gold-labeled mAbs

#### Colloidal gold labeling of mAbs

According to Liu et al. (2020), colloidal gold was prepared by the trisodium citrate method. Subsequently, gold-labeled antibodies were prepared as follows: 10 μL of ultrapure water was added to wells 1–6 in microplates, followed by the addition of 10 μL of ascites fluid (diluted at a ratio of 1: 10 with ddw) to well 1 and consecutively double-diluted to well 6. Then, 125 μL of colloidal gold solution at pH 9.0 was placed in each well, thoroughly mixed, and left standing for 5–15 min at room temperature. Next, 125 μL of 10% NaCl solution was added per well and allowed to react for 10 min at room temperature in order to observe any color changes in the solutions until the color of the solution does not change, and the concentration corresponding to the highest dilution of the well is the most suitable labeled concentration.

We added the optimal labeled amount of ascites to 1 mL of colloidal gold solution, thoroughly mixed, and allowed to react at room temperature for 15 min. Then, 100 μL of a sodium borate solution containing 20 mmol/L concentration and 10% BSA was added, mixed well, and incubated at room temperature for 5 min. The mixture was then centrifuged at a speed of 15,000 r/min for 30 min at a temperature of 4°C, followed by careful removal of the supernatant. The pellet was resuspended in boric acid buffer containing 1% (w/v) BSA. Finally, the prepared gold-labeled antibody was stored at a temperature of 4°C.

#### Screening of the strip paired mAbs

Among the positive clones, two mAbs with the highest binding affinity to the PRV gE protein were selected. The following steps were performed: each of the nine mAbs were used for detection and capturing of antibody (gold-Labeled) to each other, and a blank strip was used in a “sandwich method” mode to screen paired antibodies. A volume of 0.3 μl of the detection antibody was added dropwise onto the test strip's detection membrane and dried, and then 1 μl of the gold-labeled antibody was added dropwise onto the conjugate pad. The test strip was placed in 100 μL of the positive sample solution. The color intensity on the nitrocellulose membrane was used to determine the pairing of two specific antibodies. Finally, the characteristics of the obtained mAbs were evaluated.

#### Preparation of the immunochromatographic strip

A nitrocellulose (NC) membrane and three pads (sample pad, conjugation pad, and absorption pad) composed the test strip. The NC membrane was spotted with mAb-12E9E7 as the detection line and staphylococcal protein A (SPA) as the control line. Subsequently, it was dried at 42°C for 4 h. To prepare the sample pad, a glass wool strip was soaked in PBS (pH 8.0) containing 0.1 mol/L NaCl, 0.2% Tween-20 (v/v), and 0.1% (w/v) NaN_3_ for 30 min and dried in a drying oven at 50°C for 30 min. Next, gold-standard mAb was sprayed on glass wool at a concentration of 1 μg/μL to make a conjugate pad that was also dried at 50°C for 30 min. Following Li et al. (2021), we assembled the NC membrane, conjugate pad, fiberglass sample pad, and absorption pad sequentially on the support board, with each overlapping by 1–2 millimeters before cutting them into pieces measuring 2.79 mm by using a CM4000 cutter from Bio-Dot to form an immunochromatographic strip, which we sealed in tin foil bags along with desiccants and then stored at 4°C.

### Evaluation of PRV rapid test strips

#### Specificity evaluation of the strip

The specific sample tray comprised PRV cell cultures, PRV gE protein, and other glycoproteins derived from the same virus strain, including PRV gB, gC, and gD glycoprotein, as well as HSV-derived gE glycoproteins. Additionally, it included virus strains such as VZV, PEDV, SVA, CSFV, PRRSV, and PPV. Negative controls were represented by media such as PBS and DMEM. The identification was performed using PRV antigen dipsticks.

#### Sensitivity evaluation of the strip

The PRV standard plasmid was diluted sequentially into nine gradients, namely, 10^9^, 10^8^, 10^7^, 10^6^, 10^5^, 10^4^, 10^3^, 10^2^, and 10^1^ copies/μL, which were used as templates for real-time fluorescence RT-PCR detection of porcine pseudorabies virus, and the standard curve of real-time fluorescent RT-PCR was plotted to obtain the standard equation for the PRV real-time fluorescence RT-PCR detection method. The copy number of PRV HeNL/2017 cell culture stock was calculated and diluted by 2^1^, 2^2^, 2^3^, 2^4^, 2^5^, 2^6^, and 2^7^.

Meanwhile, the stock solution of the PRV HeNL/2017 cell culture was diluted by a series of gradients at 2^1^, 2^2^, 2^3^, 2^4^, 2^5^, 2^6^, and 2^7^ to form a sensitivity sample tray. The samples of the sensitivity sample tray were detected with PRV rapid test strips and real-time fluorescence RT-PCR, and the detection results were compared.

In addition, serial dilutions of the PRV gE protein produced in this study in the range of 2,000–15.63 ng/mL were used to determine the sensitivity of the bands.

#### Stability evaluation of the strip

In order to determine the stability of the test strip, we sealed the test strip and placed it in a 37-degree incubator for accelerated testing. These strips were stored at 37°C for 1 month and tested to verify the sensitivity of the PRV gE protein generated in this study, and its stability was determined based on the sensitivity assay results.

#### The coincidence rate of the test strip

Twenty-eight samples of spleen or lymphoid tissue of pigs were detected by using the trial PRV rapid test strip and the real-time fluorescence RT-PCR detection method of porcine pseudorabies virus, and the results of the real-time fluorescence RT-PCR detection method were used to evaluate the consistency rate with the real-time fluorescence RT-PCR detection method for porcine pseudorabies virus.

## Results

### Expression and purification of PRV glycoprotein gE

**Figure 1:**
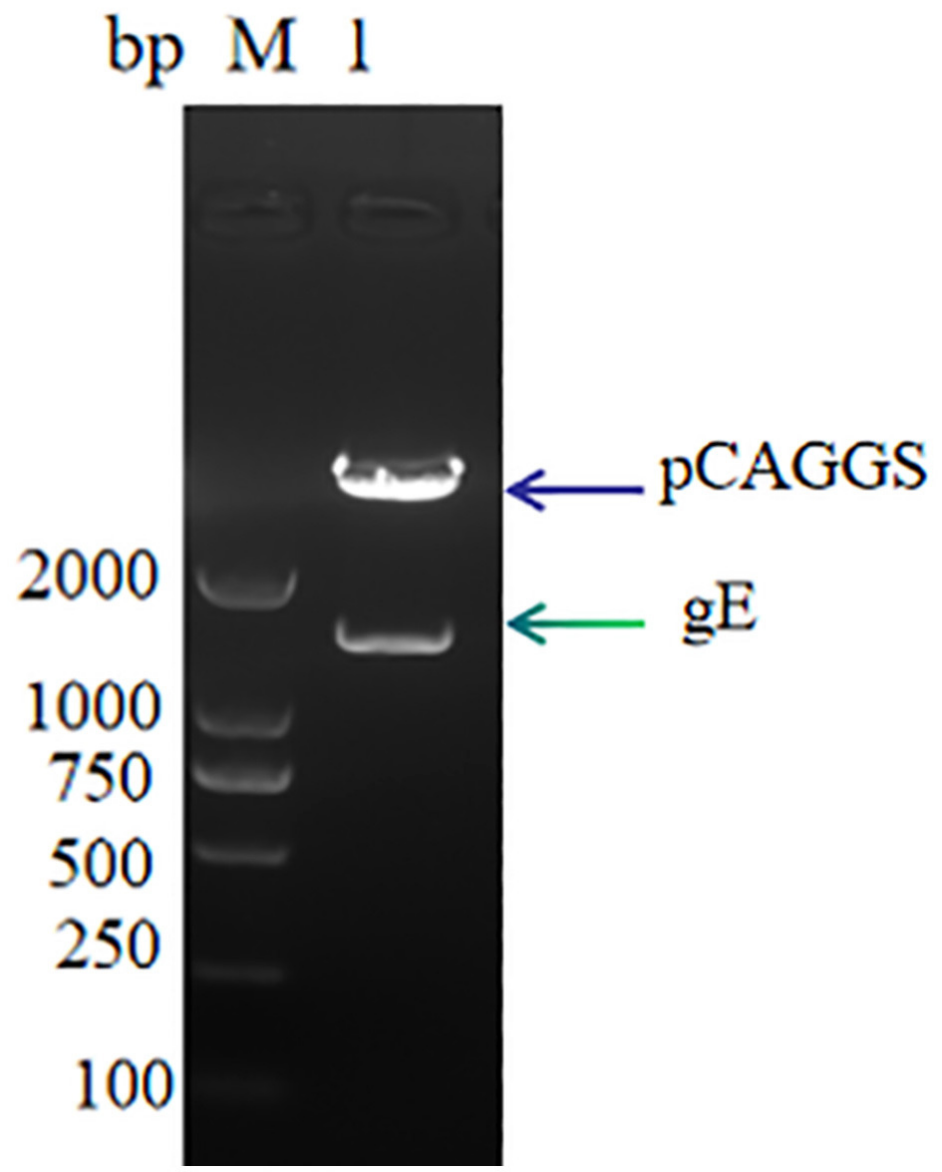
pCAGGS-gE recombinant vector digestion validation M: DL 2000, Lane 1: pCAGGS-gE digestion product.

The linearized gE target gene sequence was ligated to the pCAGGS vector sequence using T4 DNA ligase, and the recombinant expression plasmid pCAGGS-gE was obtained. The results of 1% nucleic acid electrophoresis verified by double digestion were successfully obtained, the recombinant expression vector was successfully obtained, and the size was consistent with expectations (Figure 1).

**Figure 2:**
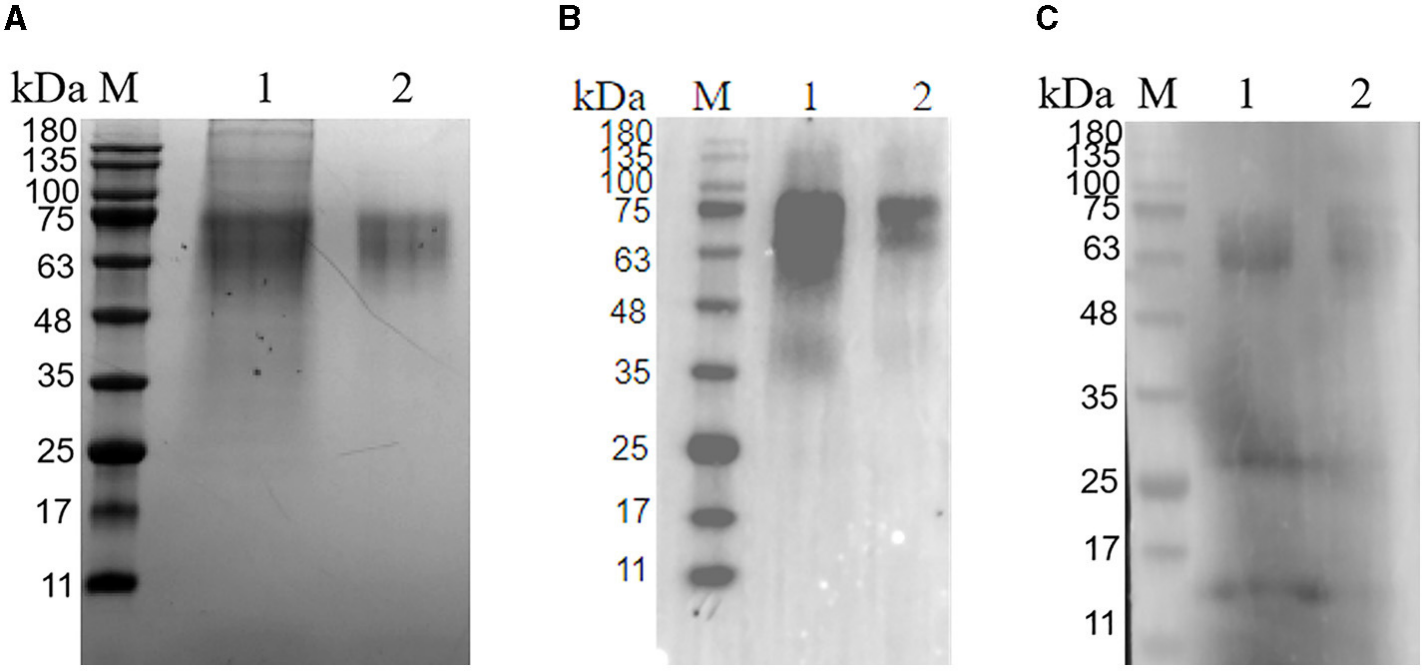
Purification of glycoprotein gE. **(A)** SDS-PAGE of the purified PRV gE protein; M: protein molecular weight marker; Lane 1: gE protein purified by A6XL; Lane 2: gE protein purified by a molecular sieve; **(B)** Western blot of the purified gE recombinant protein reacted with the His tag Antibody; M: protein molecular weight marker; Lane 1: gE protein purified by A6XL; Lane 2: gE protein purified by a molecular sieve; **(C)** Western blot of the purified gE recombinant protein react with PRV-positive field serum. M: protein molecular weight marker; Lane 1: gE protein purified by A6XL; Lane 2: gE protein purified by a molecular sieve.

The PRV gE recombinant protein was successfully expressed and purified from the mammalian expression system. The cell supernatant was centrifuged and purified to harvest the recombinant protein. As demonstrated in Figure 2, the protein eluted in PBS had a purity above 95% (Figure 2A). Western blot analysis confirmed that the gE recombinant protein could react with Anti-His tag Antibody (HRP) (Figure 2B) as well as PRV-positive field serum at a dilution of 1: 500 in 5% skim milk (Figure 2C).

### Preparation of mAbs and screening of paired antibodies

#### Screening and identification of mAbs

**Figure 3:**
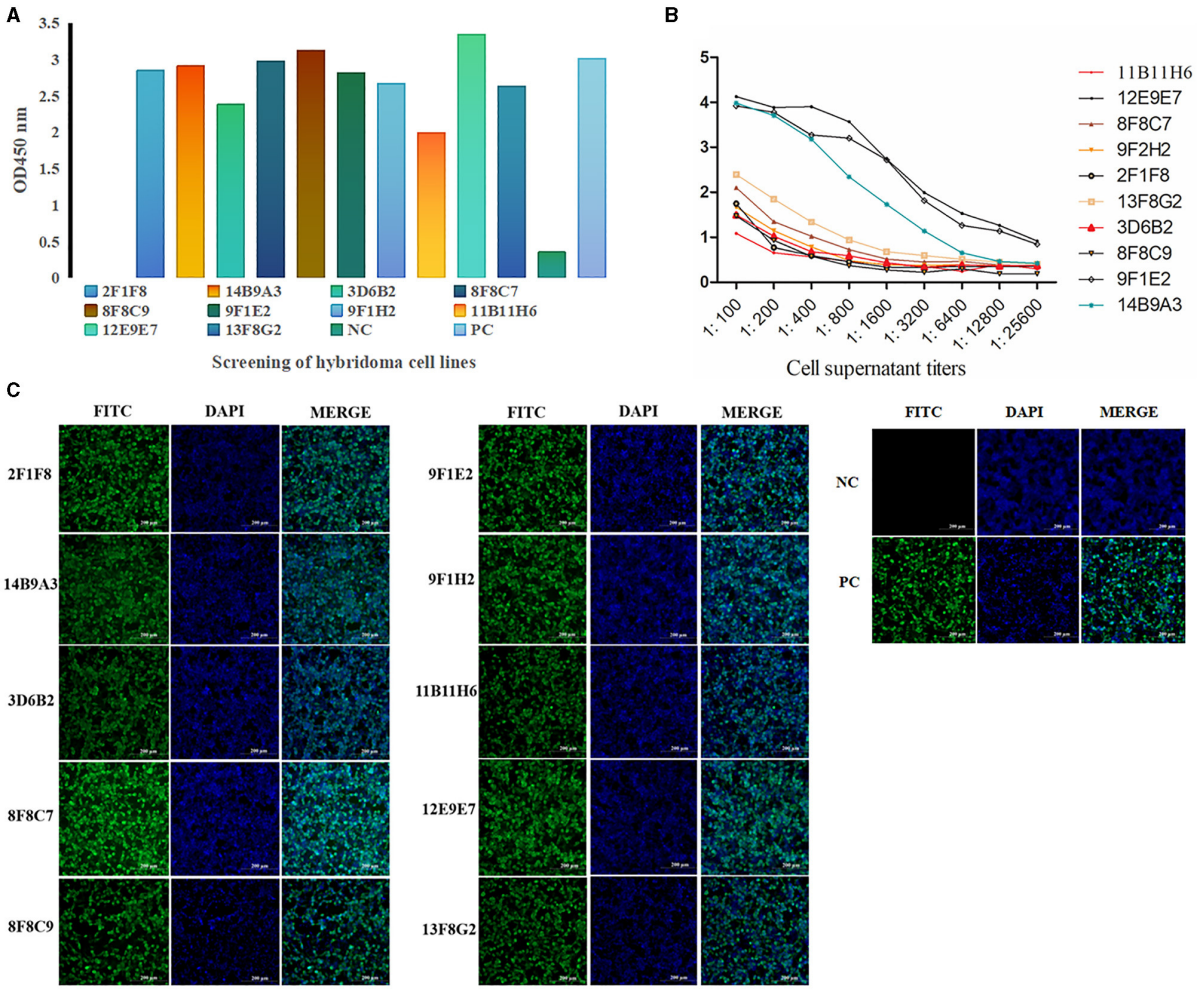
Screening and identification of mAbs. A total of 10 positive hybridoma cell lines were screened by ELISA **(A)** (the color of the column represents different cell lines), and the titers of the mAb cell supernatant ranged from 1:3,200 to 1:25,600 **(B)**. IFA demonstrated that all ten mAbs generated in this study had strong reactivity with the PRV gE protein. **(C)** IFA of the reactivity between mAbs and gE recombinant protein in 293T cells. The green color represents anti-gE mAbs, and the blue color represents nuclei (scale bars, 200 μm). These results demonstrated that mAbs react with the eukaryotic expression of the gE recombinant protein, which was consistent with the positive control.

After cell fusion, a total of 10 positive hybridoma cell lines were screened (Figure 3A), and the titers of the mAbs cell supernatant ranged from 1:3,200 to 1:25,600 (Figure 3B). IFA demonstrated that all ten mAbs generated in this study had a strong reactivity with the PRV gE protein (Figure 3C).

#### Screening of paired antibodies

**Figure 4:**
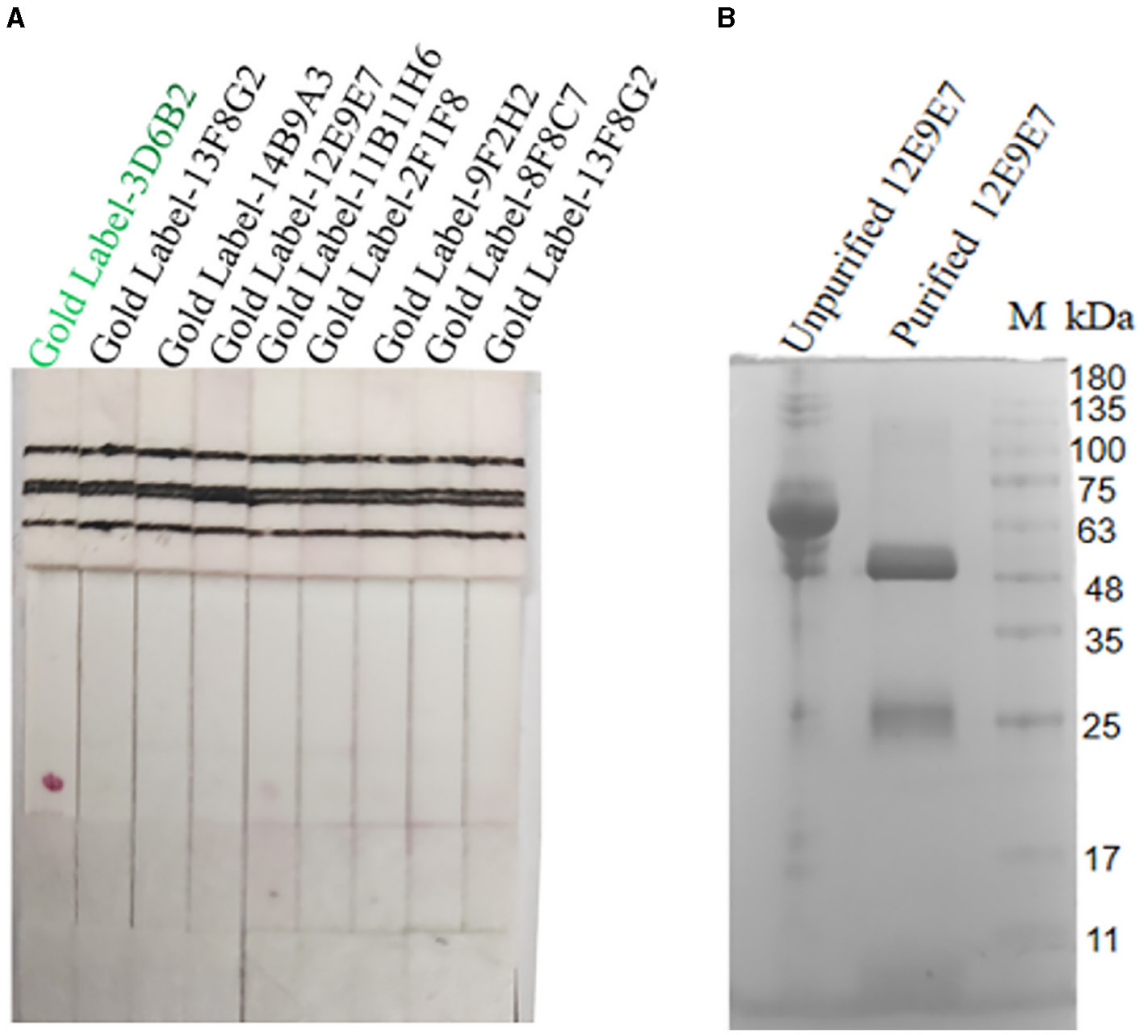
Screening of paired antibodies. Nine cell strains with high supernatant titers were selected to prepare ascites for colloidal gold labeling. When the NC film was dotted with 12E9E7, and the gold-standard 3D6B2 was used as a conjugate to bind the antigen, resulting in color development at the T line **(A)**, and the ascites fluid of 12E9E7 was subjected to SDS-PAGE analysis before and after purification **(B)**.

Nine cell strains with high supernatant titers were selected to prepare ascites for colloidal gold labeling. Antibody pairing experiments indicated that the mAb-3D6B2 as the capture antibody and mAb-12E9E7 as the detection antibody showed optimal performance according to the color development in this assay (Figure 4A). Subsequently, the capture antibody 12E9E7 was purified for the preparation of immunochromatography strips (Figure 4B).

#### The characteristics of mAbs

**Figure 5:**
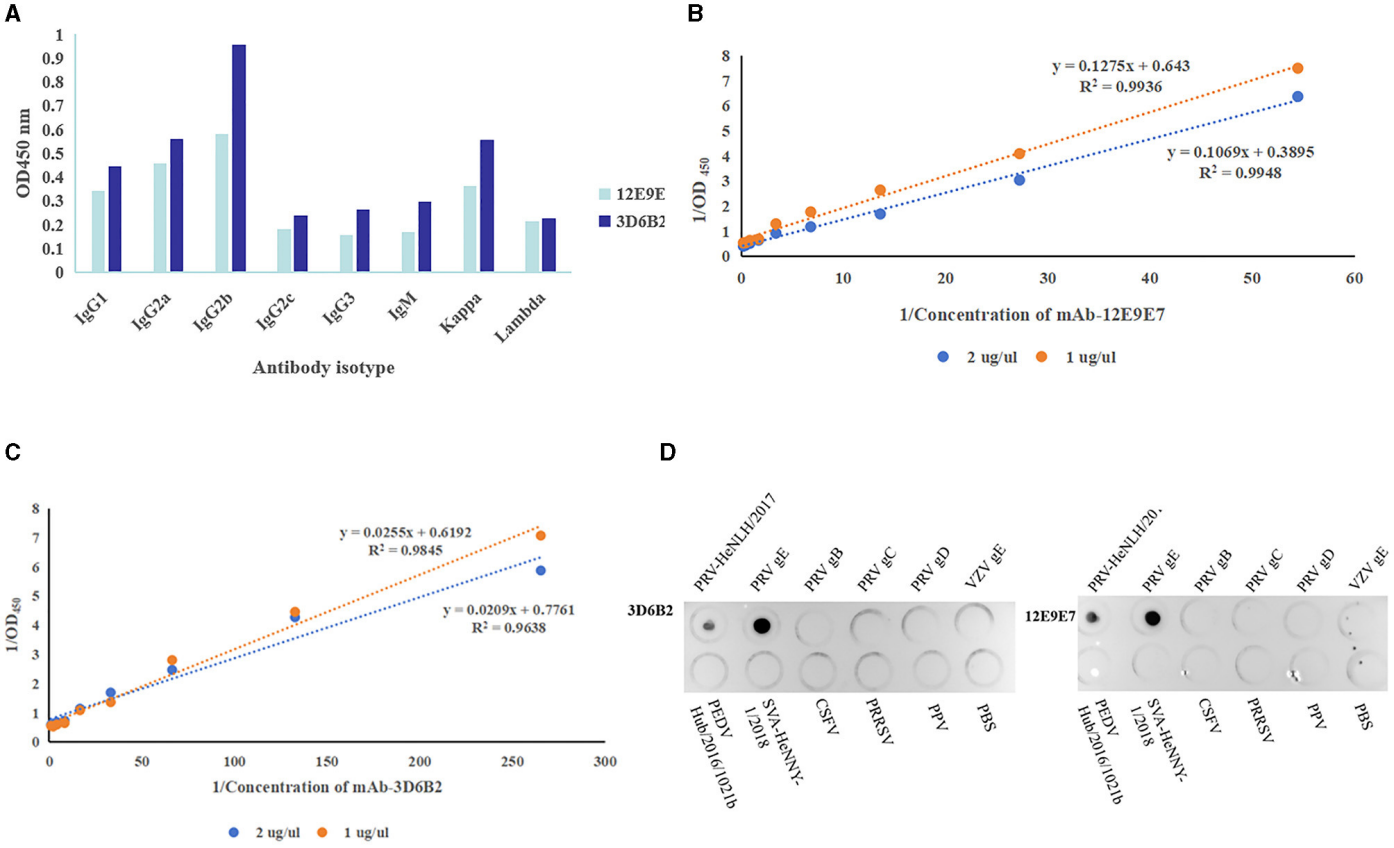
Characteristics of mAbs. **(A)** Antibody isotype identification (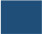 3D6B2, 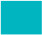 12E9E7). **(B, C)** Monoclonal antibody affinity assay. **(B)** Affinity curves of mAb-12E9E7 exhibited high correlation coefficients (the *R*^2^ value 0.9948). **(C)** mAb-3D6B2 affinity curves (the *R*^2^ values ranged from 0.96 to 0.98). **(D)** Specificity evaluation of mAb-3D6B22 and mAb-12E9E7 with (in that order) PRV cell cultures, PRV-gE proteins, PRV-gB proteins, PRV-gC proteins, PRV-gD proteins, VSV-gE protein, as well as PEDV, SVA, HSV, CSFV, PRRSV, PPV cell cultures, and PBS. The mAbs specifically reacted to PRV and gE recombinant proteins, whereas they had no response to the other proteins or diseases.

In order to gain a more comprehensive understanding of the characteristics of mAbs, we used the mouse monoclonal antibody isotyping Elisa kit (Proteintech, Wuhan) following the manufacturer's instructions to determine the subtypes of mAbs. The optical density reading at 450 nm indicated that both mAbs were classified as IgG2b and Kappa (Figure 5A). The affinity of the two mAbs was 9.994 × 10^7^ L/moL (12E9E7) (Figure 5B) and 5.69 × 10^8^ L/moL (3D6B2) (Figure 5C), respectively. Additionally, in order to determine the specificity of mAbs, PRV cell cultures, PRV-gE proteins, PRV-gB proteins, PRV-gC proteins, PRV-gD proteins, VSV-gE protein, as well as PEDV, SVA, HSV, CSFV, PRRSV, PPV cell cultures, and PBS were simultaneously detected using Dot-ELISA. The results demonstrated that the mAbs specifically reacted to PRV cell cultures and gE recombinant proteins, whereas they had no response to the other PRV proteins or diseases (Figure 5D).

#### Establishment of a rapid detective immunochromatographic strip

The colloidal gold conjugation of 3D6B2 was dispensed on fiberglass pads as conjugated mAb. The mAb 12E9E7 (1 mg/mL) was sprayed on the NC film to form a detection line. Then, the SPA was diluted to 0.5 mg/mL in PBS as the control line. The two specific mAbs of PRV detected PRV by a double-antibody sandwich mode.

#### The specificity of the test strip

**Figure 6:**
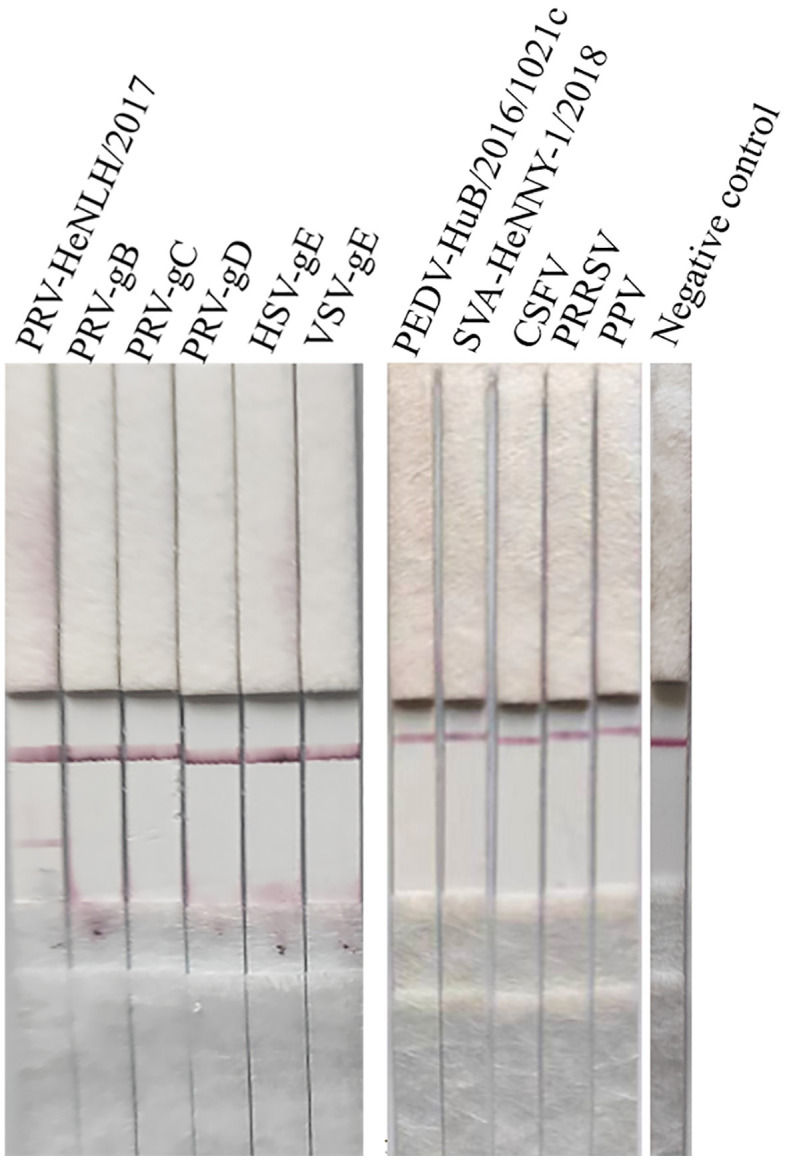
Specificity evaluation of the strip. The specific sample tray was detected by PRV rapid test strips, and the results are depicted in this figure, and only when detecting PRV cell cultures, both the T line and C line of the PRV rapid test strip were colored. In contrast, other proteins or diseases as well as the negative control displayed no coloration in the T line but showed color development in the C line.

The specific sample tray was detected with PRV rapid test strips, and the results are shown in Figure 6; only when detecting PRV cell cultures, the T line and C line of the PRV rapid test strip were colored, and the cell culture medium for detecting PRV-gB, PRV-gC, PRV-gD, HSV, VSV PEDV, SVA, CSFV, PRRSV, PPV, and negative control showed T line no color and C line color development results, indicating that the PRV rapid test strip had good specificity.

#### Real-time fluorescent RT-PCR detection method for PRV

**Figure 7:**
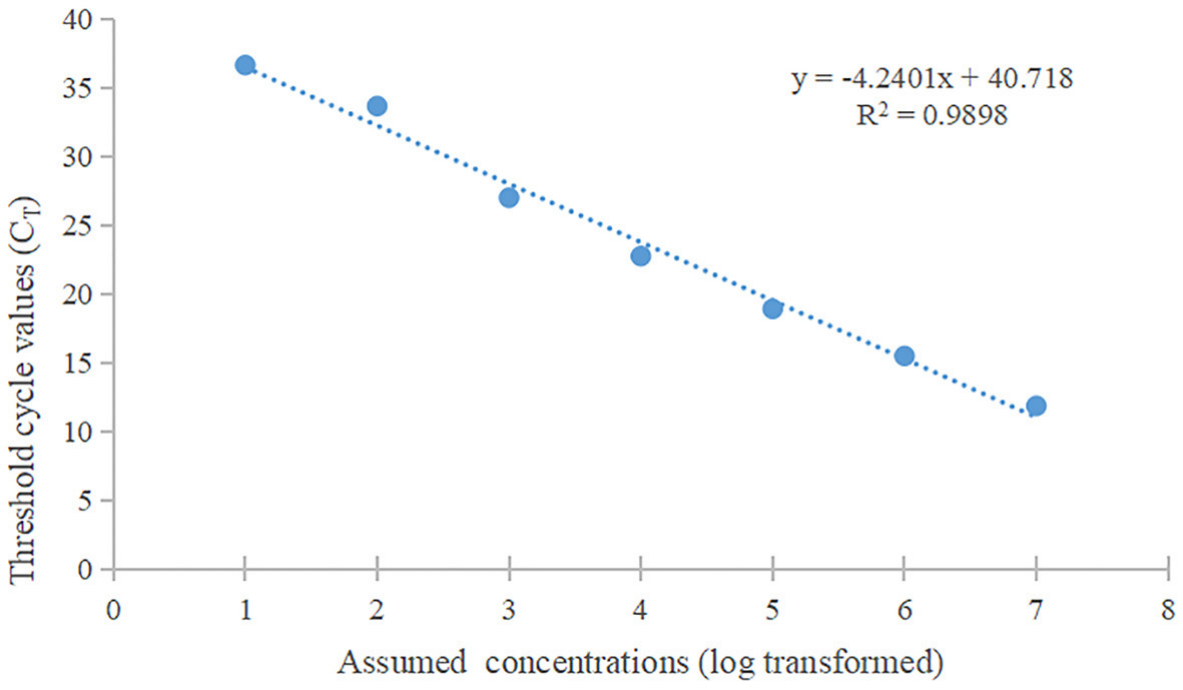
The standard curve of qPCR. The slope of this linear fitting equation is 0.9898.

The standard curve was plotted using PRV real-time fluorescence RT-PCR detection with the gradient-diluted PRV standard plasmid as a template. The equation of the standard curve was determined to be y = −4.2401x + 40.718 (*R*^2^ = 0.9898) (Figure 7). When the Ct value was >34.0, it indicated a negative test result, corresponding to a viral copy number below 38 copies/μL, thus establishing the limit of detection for the RT-PCR assay at 38 copies/μL.

### Sensitivity evaluation of the strip

#### Limit of detection of the gE protein in test strips

**Figure 8:**
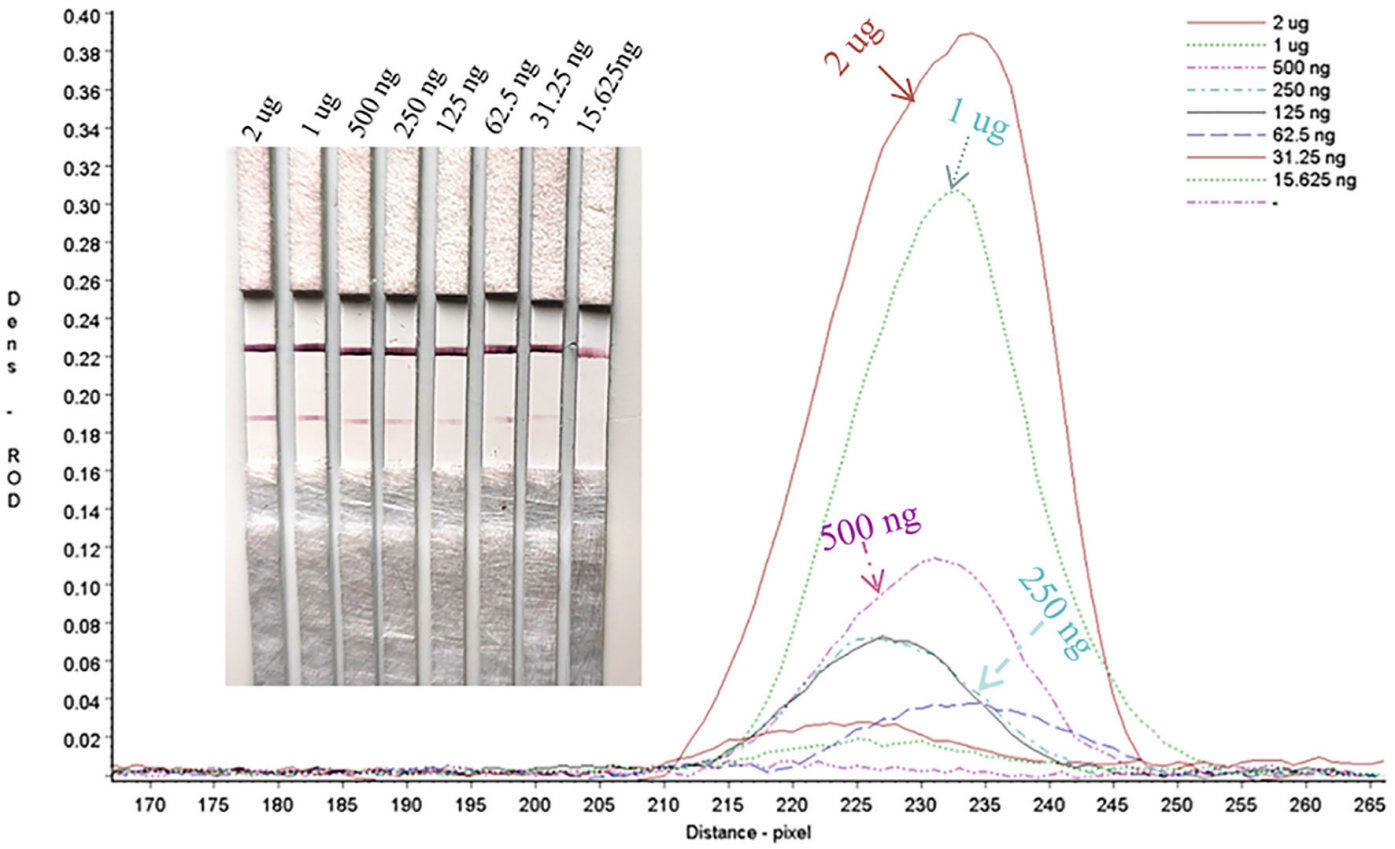
Sensitivity assay for gE protein detection.

Serial dilutions of the PRV gE protein produced in this study ranging from 2,000 to 15.63 ng/mL were used to determine the sensitivity of the strip. The G/D × area-ROD values reduced as the protein concentration in the samples reduced, so the results showed that the detection limit of RBD was 31.25 ng/mL (Figure 8; Table 1).

**Table 1:** Sensitivity evaluation of the strip.

**Region**	**Target**	**Dens—ROD**	**G/dens—ROD**	**G/DxA—ROD – pixel**	**G/pos—pixel**	**G/width—pixel**
2 μg	1	0.4729	0.1878	477.4391	230	41
1 μg	1	0.4845	0.1269	322.4699	230	41
500 ng	1	0.4112	0.0478	121.472	230	41
250 ng	1	0.4091	0.0296	75.1328	230	41
125 ng	1	0.3979	0.0287	72.9479	230	41
62.5 ng	1	0.4174	0.0182	46.2436	230	41
31.25 ng	1	0.3895	0.0152	38.5618	230	41
15.625 ng	1	0.4206	0.0096	24.3839	230	41
–	1	0.4184	0.0026	4.5934	230	41
Mean		0.4248	0.0518	131.4716	230	41
SD		0.0577	0.0631	160.5984	0	0

#### The limit of detection of PRV by the test strip

**Figure 9:**
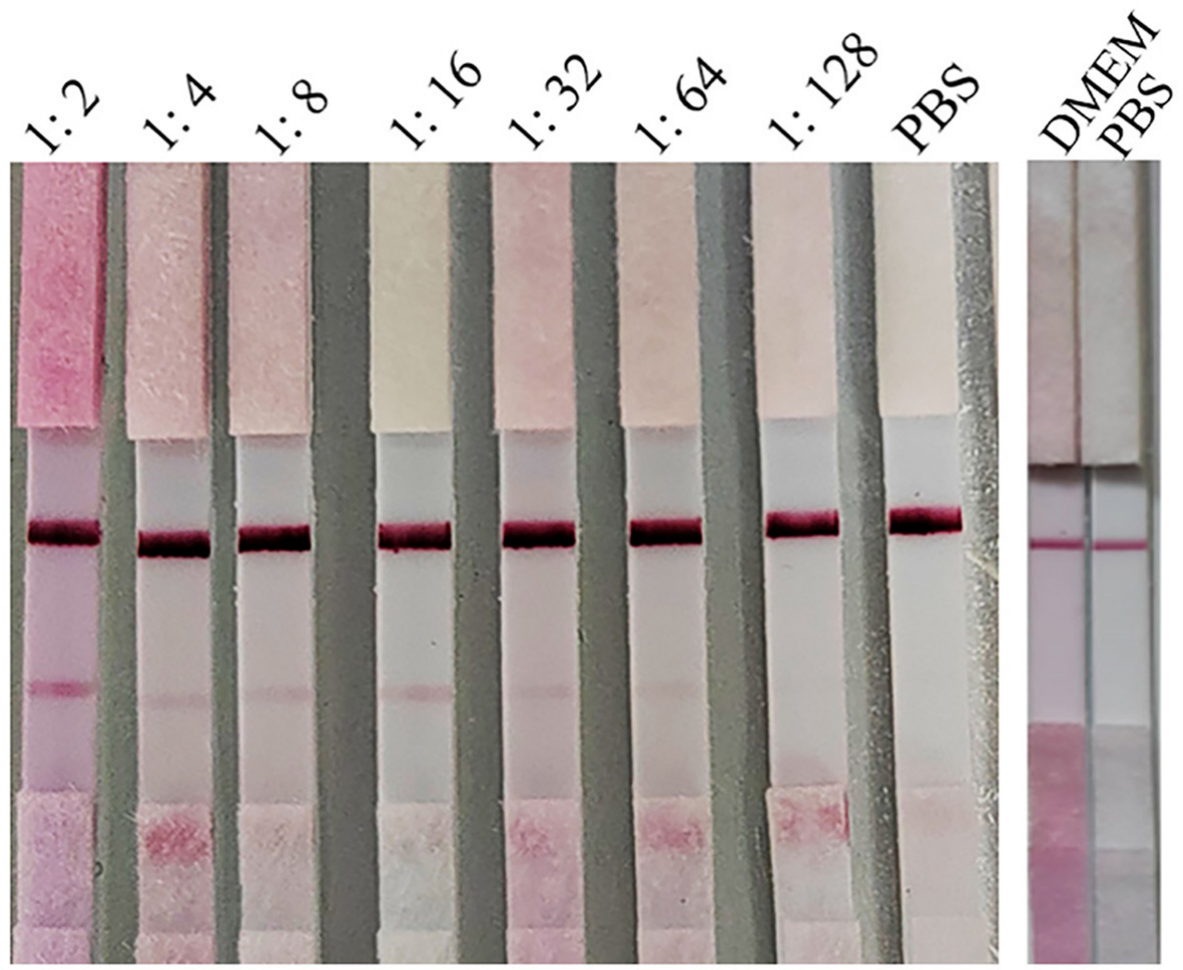
Sensitivity assay for PRV detection.

The PRV HeNL/2017 cell culture stock was diluted and then detected with PRV rapid test strips and real-time fluorescent RT-PCR detection methods. The result is shown in Figure 9. The rapid test strip had a detection limit of 1: 64 and a corresponding viral load of 1.336 × 10^3^ copies/μL (Table 2). Therefore, the number of detected copies of the PRV rapid test strip was 1.336 × 10^3^ copies/μL.

**Table 2:** Numbers of copies of gradient dilutions of PRV cell cultures.

**Dilution**	**1: 2**	**1: 4**	**1: 8**	**1: 16**	**1: 32**	**1: 64**	**1: 128**
Ct value	20.781	21.401	22.277	23.697	25.312	27.464	28.91
Number of copies	5.035 × 10^4^	3.597 × 10^4^	2.234 × 10^4^	1.033 × 10^4^	4.602 × 10^3^	1.336 × 10^3^	0.603 × 10^3^

#### The stability of the test strip

**Figure 10:**
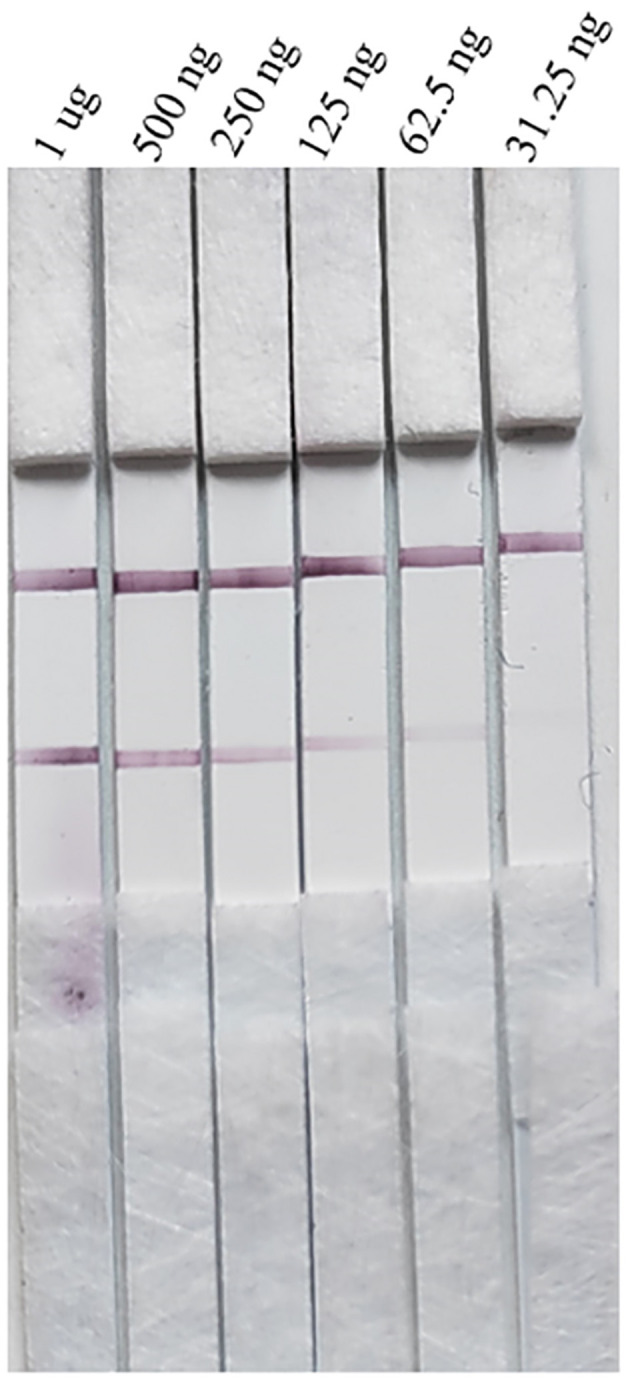
Stability evaluation of the strip.

The strips still had the same detection limit (31.25 ng/mL) for the PRV gE protein produced in this study after 1 month of storage at 37°C, indicating that the strip had good stability (Figure 10).

#### The coincidence rate of the PRV rapid test strips

Twenty-eight samples of spleen or lymphoid tissue from pigs were detected by PRV rapid test strip and real-time fluorescence RT-PCR. The results are shown in Table 3; 15 samples were found positive for porcine pseudorabies virus by the real-time fluorescence RT-PCR test method, and 14 samples were found positive for PRV rapid test strips. Based on the detection results of the real-time fluorescence RT-PCR detection method for porcine pseudorabies virus, the coincidence rate of PRV rapid test strips was 96.4% (27/28).

**Table 3:** Detection twenty-eight samples of spleen or lymphoid tissue from pigs.

	**Porcine pseudorabies virus real-time fluorescence RT-PCR detection**			
		**Positive**	**Negative**	**Total**
PRV rapid test strips	Positive	14	0	14
	Negative	1	13	14
	Total	15	13	28

## Discussion

As a potential zoonotic disease, PRV poses a threat to both the breeding industry and public health security (Ai et al., 2018; Wang et al., 2020). Since 2011, the circulation of wild-type PRV strains in China's pig farms has brought significant challenges to the pig industry and the prevention and control of epidemic diseases (An et al., 2013). To control these domestic circulating variants, researchers have developed a gene deletion vaccine with the PRV variant as its parent strain. This vaccine can provide complete immune protection efficacy in pigs and represents an ideal candidate for a PRV variant. In order to enhance the control and prevention of PRV in China, mandatory vaccination is accompanied by virological and serological surveillance as well as stringent biosecurity procedures. It is imperative to intensify nationwide testing and strengthen monitoring of infections in animals other than pigs. Although conventional methods for detecting PRV such as ELISA, RT-PCR, qPCR (Deblanc et al., 2019; Cheng et al., 2021), and rabbit inoculation test are accurate, they do have certain limitations. For instance, the PCR technology requires advanced skills and may not be suitable for routine farm testing; rabbit inoculation tests require professional personnel and are not suitable for large-scale animal husbandry; ELISA based on antibodies in the sample may miss the optimal period for prevention and control. Although the new labeled materials such as colloidal selenium and up-converting phosphor nanoparticles were abundant (Li et al., 2011), we still chose the conventional colloidal gold in this study because colloidal gold is more stable, sensitive, and specific. The test results are visible to the naked eye and imply short time consumption, high sensitivity, good specificity, and suitability for grassroots promotion and application (Li et al., 2015, 2023).

In this study, we selected the PRV gE protein as our research target for the following reasons: first, the gE protein is a glycoprotein located in the viral envelope, which is commonly used to detect wild-type virus infection, and its antibody detection can also distinguish between wild-type virus infection and vaccine immunization animals (Nauwynck, 1997; Ao et al., 2003). Additionally, establishing methods to detect the gE protein allows for strict quality control of inactivated vaccines and live vaccines lacking gE. It has been reported that the PRV gE protein has successfully been expressed in *E. coli*, yeast, and insect cells. The established ELISA, immunofluorescence, and other methods can effectively distinguish wild-type virus infection from vaccine-induced immune antibodies, thus demonstrating promising application prospects. However, some conventional challenges persist: most of the gE recombinant proteins expressed by the prokaryotic expression system exist as inclusion bodies with low antigen activity, which often fails to meet clinical detection requirements (Wu et al., 2023). There is low expression in yeast expression systems (Ren, 2004) and long expression cycles in the insect system (Zhu et al., 2019). All of these factors significantly impact the practical application of the PRV gE protein. The HEK 293F expression system modifies the expressed protein to ensure correct folding, high levels of protein expression, and preservation of a more complete natural conformation with enhanced biological activity (Nettleship et al., 2015; Qiao-Li et al., 2019). In this study, we used 293F cells for expressing the PRV gE protein. The size of the obtained gE recombinant protein was consistent with Lang et al.'s findings, while achieving higher purity than found in previous studies. These results provide valuable insights for developing detection methods.

In this study, a total of 10 cell lines producing mAbs against PRV gE were generated. Nine strains were selected for ascites production, while one strain exhibited low supernatant titers, possibly due to a non-dominant region of antigenic determinants recognized by the cell lines. Two mAbs, namely, 12E9E7 and 3D6B2, were selected as detection and capture antibodies in the sandwich detection mode for the immunochromatographic test strip. The affinity of the two mAbs was determined to be 9.994 × 10^7^ L/moL (12E9E7) and 5.69 × 10^8^ L/moL, respectively. Notably, the high-affinity mAb-3D6B2 was used as capture antibodies in accordance with established principles. Furthermore, it is worth mentioning that two mAbs exhibited no cross-reactivity toward other PRV proteins and viruses, thereby demonstrating their exceptional specificity. The remaining eight mAbs can be used for fundamental research in the field of immunology, pathogen detection, antigen purification, disease diagnosis, and prevention (Liu et al., 2015). In this study, a rapid immunochromatographic test strip was developed for detection of the PR antigen using the high-affinity mAb-3D6B2 along with the highly sensitive mAb-12E9E7. Gradient-diluted PRV HeNL/2017 cell cultures were tested with a positive limit dilution of 1:64 and a viral load of 1.336 × 10^3^ copies/μL. The test strip had good specificity and showed no cross-reactivity with other viral antigens. Upon comparing the results of the test strips, it was observed that 15 samples tested positive for porcine pseudorabies virus by use of the real-time fluorescence RT-PCR detection method, while 14 samples were detected by the test strips. By analyzing these findings, it can be speculated that this discrepancy may be attributed to the sensitivity of the porcine pseudorabies virus real-time fluorescence RT-PCR assay, which has a limit of detection lower than 1.336 × 10^3^ copies/μL and may yield false-negative results for samples with low viral loads. It is possible that the sensitivity of the test strip is slightly lower when compared to RT-PCR; however, its higher coincidence rate (27/28) with RT-PCR results indicates that the PRV rapid test strip possesses characteristics such as convenience, speediness, high sensitivity, and high specificity. Therefore, it can be effectively utilized for rapid diagnosis of clinical cases involving PR.

Studies have demonstrated that since 2016, the prevalence of PRV epidemics in our country has continued to be predominantly driven by variants. The comparison of gE amino acid sequences revealed individual site mutations compared to the classical strain, while no specific mutations were observed when compared to variant strains (Ujvári et al., 2019). The monoclonal antibody used in this study is a variant-specific monoclonal antibody capable of distinguishing between the wild-type and vaccine antigens. Despite variations in the amino acid sequences of PRV variants and classical strain gE antigens, their sequence homology remains remarkably high. However, it should be noted that the monoclonal antibody employed in this study cannot differentiate between PR classical strain and variant strain antigens, which highlights an area for future research.

PRV primarily manifests as a latent infection, wherein the vaccinated herd may resume normal production but still experience reactivation of hidden infections and subsequent clinical symptoms under stress. This makes them a secondary source of long-term poison and dispersion, perpetuating the circulation of the disease within the pig population for an extended period. Consequently, it significantly impedes the development of China's pig industry. Therefore, conducting research on preventive and control measures for swine pseudorabies is crucial. The immunochromatography test strip developed in this study offers a valuable diagnostic tool for treating PR disease.

## Conclusion

In this study, we used the HEK293F expression system to obtain a high-purity gE recombinant protein, which provided experimental materials for the study of the structure and function of the PRV gE protein. The PRV gE protein was used to immunize mice, resulting in the production of highly sensitive and specific mAbs 12E9E7 and 3D6B2. These mAbs were then used in the development of an immunochromatographic test strip for rapid detection of PRV. This alternative method, based on labeled monoclonal antibody–monoclonal antibody interception, exhibits both high sensitivity and specificity, making it a valuable substitute for real-time fluorescence RT-PCR testing in diagnosing PR clinical diseases.

## Data availability statement

The original contributions presented in the study are included in the article, further inquiries can be directed to the corresponding author.

## Ethics statement

The animal study was approved by the Key Laboratory of Animal Immunology, Henan Academy of Agricultural Sciences, China, in line with its policies and procedures (LLSC100166). The study was conducted in accordance with the local legislation and institutional requirements.

## Author contributions

JY: Data curation, Visualization, Writing – original draft, Writing – review & editing. HuL: Conceptualization, Methodology, Validation, Writing – review & editing. YC: Investigation, Validation, Visualization, Writing – review & editing. JZ: Investigation, Visualization, Writing – review & editing. YL: Formal analysis, Software, Writing – review & editing. ZL: Conceptualization, Investigation, Methodology, Visualization, Writing – review & editing. XZ: Supervision, Writing – review & editing. HoL: Validation, Writing – review & editing. PD: Validation, Writing – review & editing. EL: Validation, Writing – review & editing. YZ: Writing – review & editing. SW: Writing – review & editing. AW: Project administration, Visualization, Writing – review & editing.
